# Cancer Research

**Published:** 1904-07-16

**Authors:** 


The Hospital.

A Journal of

The Medical Sciences and Hospital Administration.

Vol. XXXVI.—No. 929. JULY 16, 1904.

### Cancer Research.

The report presented by the Executive Committee of the (now) Imperial Cancer Research Fund, and which was received by the Prince of Wales and the General Committee on the 8th instant, describes a very hopeful commencement of a great national enterprise. In addition to extensive experimental work, inquiries into the prevalence of cancer in different races of men and in different animals have been instituted, and the data which have led to the assertion that cancer is increasing have been subjected to critical examination. The chief facts which have been ascertained or placed upon a more certain footing during the year covered by the report may be summarised as follows :—

Cancer has been discovered to pervade at least all classes of the vertebrate kingdom, to present constant characteristic features in all animals, and to fall into the same groups for classification purposes. Throughout all races of men and animals it exhibits the same predilection for certain age periods proportionate to the varying durations of the span of life in all the nearly allied and widely removed species of animals in which it occurs ; and in all animals alike cancer, *quâ* cancer, is without any characteristic symptoms. An important distinction has been established between the infectious diseases and cancer. An infectious disease, such as tuberculosis, which is common to man and certain animals, presents a general similarity in the disease itself and in the symptoms produced by the same infective agent, although both show variations from species to species. Cancer, on the other hand, while showing the same essential features in all animals, from mankind to fish, and a total absence of characteristic symptoms throughout, is not transmissible from one species to another species. The transmissibility of cancer is of a limited and peculiar nature, and merely implies that the cancer cell may continue to grow if transferred to another host of the same species. The new host, however, does not become infected with cancer, but merely provides a soil in which the transplanted cells may grow. These peculiarities prove what had long been suspected, namely, that the cancer cell itself is endowed with powers of growth which are unique. Phenomena have been observed which appear to show that the cancer cell can re-acquire the power of self-propagation. If future work should confirm the last observation the problem of the continued growth of cancer cells will have been solved. The hypothesis that cancer is an infectious disease may be said to have been invalidated by the import of some of the foregoing observations. The identity in the appearance of cancer in all animals, the absence of characteristic symptoms, and the impossibility of transferring it from one species to another, demonstrate that the same infective agent cannot be the cause of cancer in man and in animals. The fact that in the successful transmission of cancer it is the cancer-cell itself which grows, and that it can only grow in animals of its own species, excludes altogether a parasitic causation. The comparative study of the disease in all its features and in all animals has done much towards establishing a common biological law in relation to it. It seems justifiable to conclude that its cause must be sought in those properties of cells which are the only characters common to all the animals in which cancer occurs. The nature of the transmissibility affords experimental proof of the truth of this conclusion, and observations of the minute cell structure have brought to light facts which may ultimately afford an adequate explanation of the mechanism by which the power of independent proliferation is acquired and maintained by the cells of a cancer.

Investigations are in progress with regard to the possibility of a rational serum diagnosis and serum treatment. The powers of radium as a curative agent have been examined, but have yielded no promise of general utility.

Statistical inquiries of great range and minuteness have been instituted, and the extent to which the disease pervades the animal kingdom indicates that it cannot be attributed to geographical surroundings, geological formation, climate, food, or external conditions of any kind. It is gratifying to be assured that careful analysis of the data upon which the mortality statistics of cancer have been based does not indicate any real increase of the disease.

The report contains one weighty warning. Cancer is an insidious disease, which, as such, gives no evidence of its presence, and the circumstance which first attracts attention is too often merely one of its consequences. The public and the medical profession are therefore gravely warned not to delay surgical interference in the hope that some means will be discovered by which it may be replaced or rendered unnecessary. No attitute is to be more strongly deprecated than that of procrastination, of waiting for definite evidence of the cancerous nature of a suspicious growth. By a proper appreciation of this warning the public can best assist the medical profession in the prevention or alleviation of much suffering.

